# Single-Item Measurement of Suicidal Behaviors: Validity and Consequences of Misclassification

**DOI:** 10.1371/journal.pone.0141606

**Published:** 2015-10-23

**Authors:** Alexander J. Millner, Michael D. Lee, Matthew K. Nock

**Affiliations:** 1 Department of Psychology, Harvard University, Cambridge, Massachusetts, United States of America; 2 Department of Psychology, The University of Texas at Austin, Austin, Texas, United States of America; Medical University of Vienna, AUSTRIA

## Abstract

Suicide is a leading cause of death worldwide. Although research has made strides in better defining suicidal behaviors, there has been less focus on accurate measurement. Currently, the widespread use of self-report, single-item questions to assess suicide ideation, plans and attempts may contribute to measurement problems and misclassification. We examined the validity of single-item measurement and the potential for statistical errors. Over 1,500 participants completed an online survey containing single-item questions regarding a history of suicidal behaviors, followed by questions with more precise language, multiple response options and narrative responses to examine the validity of single-item questions. We also conducted simulations to test whether common statistical tests are robust against the degree of misclassification produced by the use of single-items. We found that 11.3% of participants that endorsed a single-item suicide attempt measure engaged in behavior that would not meet the standard definition of a suicide attempt. Similarly, 8.8% of those who endorsed a single-item measure of suicide ideation endorsed thoughts that would not meet standard definitions of suicide ideation. Statistical simulations revealed that this level of misclassification substantially decreases statistical power and increases the likelihood of false conclusions from statistical tests. Providing a wider range of response options for each item reduced the misclassification rate by approximately half. Overall, the use of single-item, self-report questions to assess the presence of suicidal behaviors leads to misclassification, increasing the likelihood of statistical decision errors. Improving the measurement of suicidal behaviors is critical to increase understanding and prevention of suicide.

## Introduction

Suicide is a leading cause of death in the United States and throughout the world [1]; however, progress understanding and preventing suicide has been relatively slow. Numerous consensus articles have focused on the *definitions* of suicidal behaviors [2–4] but *measurement* has received less critical attention. One major limitation is the common use of single-item self-report measures to assess the presence of suicidal ideation, plans, and attempts (e.g., “Have you ever made a suicide attempt?”). We use the term *single-item measurement* to refer to the use of one question to assess whether a person has engaged in a single behavior (e.g., suicide attempt). Most single-item measures do not provide a clear definition of the behavior being assessed. Several commonly used instruments, such as the Youth Risk Behavior Survey (YRBS; [5]) and the World Health Organization Composite International Diagnostic Interview (CIDI; [6]) contain single-item questions to assess suicidal behaviors. Recent epidemiologic reviews on suicidal behaviors reveal that more than half of all studies on suicide use this single-item approach [7,8].

Although single-item measurement is widely used, few studies have examined whether it results in the reliable and accurate classification of self-reported suicidal behaviors. Two characteristics of single-item assessment might cause misclassification: a lack of *clarity* and a lack of *coverage*. Regarding the former, respondents may not clearly understand the specific behavior in question. Although researchers have established definitions for suicidal behaviors (e.g., a suicide attempt requires engaging in a potentially lethal behavior with some intention of dying [2,3]), single-item questions do not inform participants of this definition. Therefore, participants have to rely on their own definitions of suicidal behaviors, which could be incorrect and lead to misclassification. Regarding the latter, most single-item assessments query the presence of suicide ideation, plans, and attempts; however, there are many more subtle steps in the process of attempting suicide that are omitted when using single-item assessment (consensus articles labeling suicidal behaviors also fail to identify and define these subtle behaviors [4]). For example, many people report starting to take steps to attempt suicide but stopping themselves before acting (i.e., an aborted suicide attempt). When confronted with a single-item suicide attempt question, people that engaged in an aborted attempt may be confused about whether this action constitutes a “suicide attempt” or not and incorrectly endorse having made a suicide attempt.

Prior studies suggest that single-item assessment results in misclassification. For example, among a nationally representative sample of adults, 40% of those who endorsed a single-item measure of a suicide attempt later indicated in follow-up questions that they did not actually intend to die from their behavior [9]. In another study, 20% of those who endorsed a single-item measure of a “suicide attempt” reported in follow-up questions that they took steps towards suicide but did not actually *engage* in the potentially harmful action [10]. Misclassification has consequences in both clinical and research practice. Anecdotally, the authors have encountered clinical situations where both clinicians and patients erroneously labeled an action a “suicide attempt.” This misunderstanding could alter doctors’ perception of risk. Imprecise measurement can impede scientific progress, particularly if misclassification rates are large, potentially altering the true probability of finding a result under the null hypothesis (*p* value), increasing the probability of statistical errors.

The purpose of this study is to: (i) test whether single-item measures result in the inaccurate assessment of self-reported suicide ideation, plans, and attempts and (ii) explore the extent to which current misclassification rates can increase the probability of statistical errors. Participants were asked three single-item *gate questions* that closely resemble commonly used single-item questions assessing suicidal ideation, plans, and attempts, followed by several *follow-up questions* that assessed more specific, fine-grained outcomes. In addition, we built statistical simulations to test the consequences of the observed misclassification. We hypothesized that: (i) single-item assessment would lead to substantial group misclassification because participants actual behaviors would not meet researchers’ definitions for the behavior in question and (ii) there would be a range of suicidal behaviors beyond those assessed with single-item questions. Finally, we hypothesized that (iii) statistical simulations would reveal that the observed degree of misclassification would be associated with increased probability of false negative and false positive conclusions.

## Materials and Methods

### Participants

Participants were recruited via Craigslist postings throughout the United States from April 2013 through September 2013. Postings contained two different titles to advertise to suicidal (“Online Survey about Suicidal Thoughts and Behaviors”) and non-suicidal people (“Online Survey about Hard Times”) to capture those with a range of thoughts about death and suicide. All participants first entered their age. Participants under 18 years of age were not permitted to take the survey. Participants that were 18 or older gave informed consent through the online survey by selecting a “Yes” radio button in response to the following statement at the bottom of the informed consent form: “I have read, understood, and, if necessary, printed a copy of the above consent form. I agree to participate in this study and I understand that I am free to withdraw at any time without incurring any penalty.” Participants that selected “No” were not permitted to take the survey. All consent responses are stored digitally in the output of the survey data. Inclusion criteria were: (1) 18+ years of age and (2) ability to correctly answer three questions regarding the study to ensure comprehension of consent and safety information. Upon completion of the survey, participants could enter their email address into a $350 raffle on an independent webpage. The Harvard University Institutional Review Board approved the study, including the consent procedure.

After giving informed consent, participants were informed that (1) the purpose of the survey was to collect information and should not be considered an emergency service or a website to receive psychiatric care and (2) if they were distressed or increasingly suicidal, a link to resources and a phone number to a suicide hotline were always located at the bottom of the screen.

Of the 2,371 subjects that entered the survey, 753 were excluded from analyses because they failed to complete even one section of the survey or did not provide valid survey data. Of the remaining 1,618 participants that completed at least one section, 80.5% completed all survey questions. Table 1 contains the demographic information for these 1,618 participants.

**Table 1:** Demographic information.

Age	*M*	*SD*
	33.9	12.8
	**Median**	**Range**
	30.0	18–79
**Gender**	***N***	**%**
Female	1203	74.4
Male	398	24.6
Other	17	1.1
**Race**	***N***	**%**
Caucasian	1214	75.0
Other or Mixed Race	134	8.3
Race No Response	126	7.8
African American	107	6.6
Native American/Alaska Native	26	1.6
Native Hawaiian/Pacific Islander	11	0.7
**Ethnicity**	***N***	**%**
Not Latino	1404	86.8
Latino	180	11.1
Ethnicity No Response	34	2.1
**Location**	***N***	**%**
Northeast (10 States)	444	27.4
Midwest (11 States)	341	21.1
West (10 States)	336	20.8
Southeast (13 States)	261	16.1
Southwest (4 States)	221	13.7
Location No Response	9	0.6
Not from the U.S.	6	0.4

### Measures

#### Online Survey

**Fig 1:**
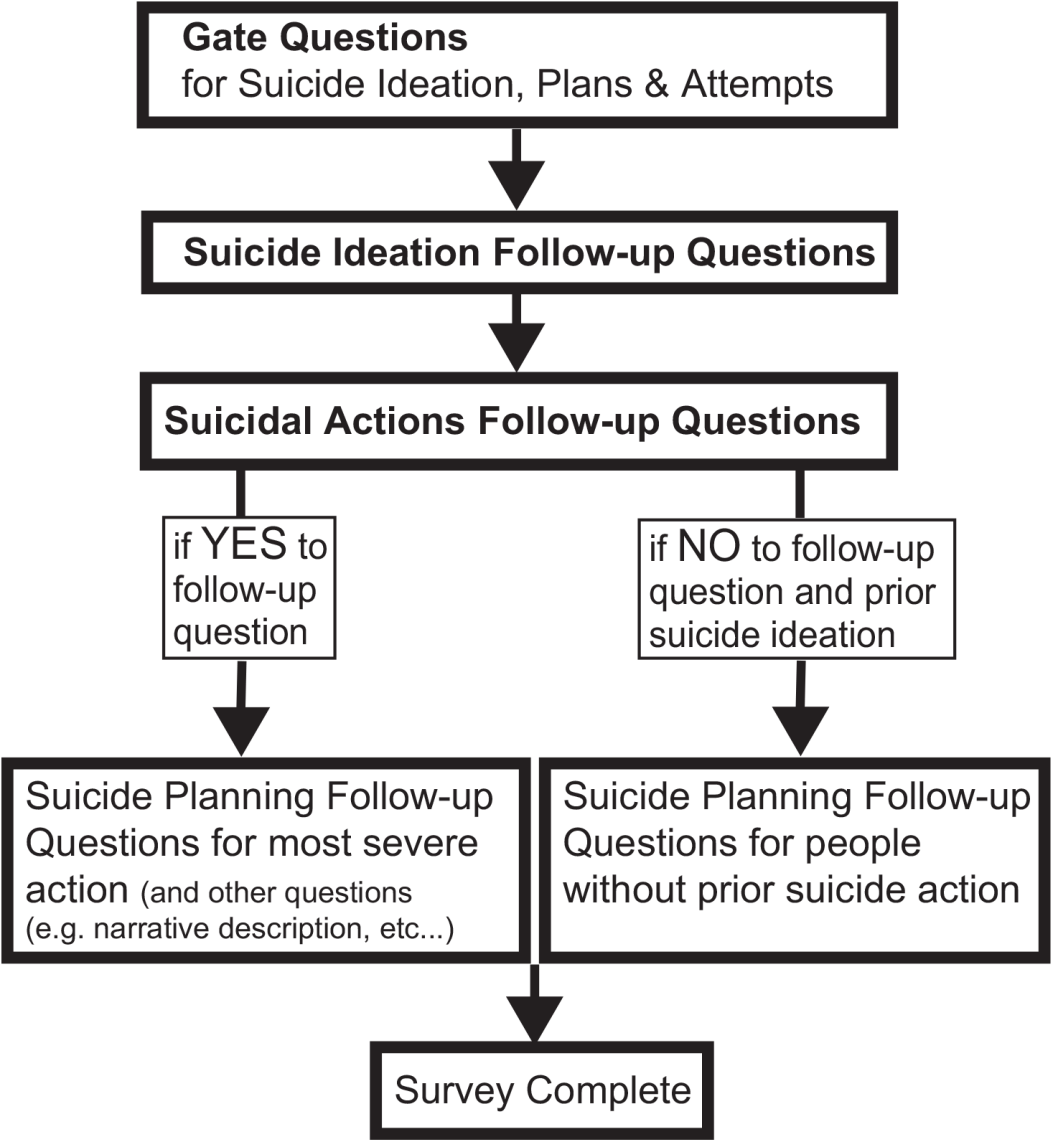
A flow chart of the online survey. Gate questions are single-item questions for three suicidal outcomes. Suicide ideation follow-up questions include eight different thoughts and suicide action follow-up questions include five different actions. Participants that denied any suicidal thoughts or behaviors in the first three sections skipped to the end of the survey. Participants that endorsed any suicidal thought or behavior completed several follow-up questions regarding planning behaviors and narrative descriptions of their behavior.

The online survey was administered using Qualtrics and data were collected anonymously and contained three required sections. In addition, participants’ responses could trigger several additional sections that included, at minimum, age of first and most recent occurrence and number of times an action occurred, where applicable. Fig 1 contains a flow chart of the survey.

Section 1 included three *gate questions*: single-item yes/no questions regarding a past history of suicide ideation, plans, and attempts. Section 2 contained suicide ideation *follow-up questions* that assessed the presence of eight different thoughts ordered in increasing severity (see Tables A-E in S1 Online Survey for precise wording). We used the follow-up questions to assess inconsistent responses among participants (e.g., test whether participants that endorse a gate question give different responses to follow-up questions or participants that endorse the same follow-up questions answer differently to a gate question). The two lowest severity items included philosophical questions about death, followed by two low severity, passive ideation items (i.e., wishing to disappear and wishing one was not born), two high severity, passive ideation questions (i.e., life is not worth living or I wish I was dead; [11]), and two active ideation questions (i.e., maybe I should kill myself or I should kill myself). Participants that endorsed any passive or active suicidal thoughts were asked additional questions about their most severe thought (results not included here).

Section 3 contained suicidal action follow-up questions that assessed the presence of five different actions including: (i) non-suicidal self-injury, (ii) suicide gesture, in which a person had no intent to die and only wanted to *look like* s/he was attempting suicide, (iii) aborted suicide attempt, in which a person started to take steps to kill herself and stopped at the last minute, (iv) interrupted suicide attempt, in which someone or something else stops a person from attempting suicide and (v) a suicide attempt, described as having engaged in a potentially lethal behavior with some intention of dying.

If participants endorsed at least one prior suicidal action, they were asked additional questions about the highest severity action (and the most recent if more than one). We also queried how much time passed between planning steps and the suicidal action.

If participants endorsed a suicidal thought during the gate questions or follow-up but did not endorse a suicidal action, they were directed to a suicide planning follow-up section. This section included questions regarding the presence or absence of having carried out any planning steps (e.g., “Have ever thought of a method or methods that you would use to kill yourself?”).

Overall, all participants answered, at minimum, Section 1 (i.e., gate questions), Section 2 (i.e., follow-up ideation questions) and Section 3 (i.e., action follow-up questions). If they endorsed ideation, they were asked additional questions about their most severe thought. If they endorsed a suicidal action, they were asked additional questions, including planning items and open-ended questions, about the most recent, highest severity action. If they endorsed suicidal ideation but not a suicidal action, they were asked about any planning steps taken. If they denied all suicidal ideation and actions, the survey terminated following Sections 1, 2, and 3.

### Coding of Narrative Descriptions

#### Suicidal Actions

To better understand exactly what occurred during any suicidal actions (i.e., during an aborted attempt, interrupted attempt, or suicide attempt), participants responded to multiple questions that required narrative descriptions (e.g., “What exactly happened?”, “Did you sustain any injuries or have any physical problems as a result?”).

Despite consensus articles [2–4], categorizing suicidal behaviors remains contentious. For the current study, to categorize a behavior as a suicide attempt, participants had to describe an event where they explicitly engaged in a potentially harmful or potentially lethal behavior, regardless of whether it was low lethality (e.g., “a few pills”) or actual harm did not occur (e.g., the trigger was pulled but the gun did not fire). Some respondents reported engaging in potentially lethal behavior with some intention of dying, but did not endorse having made a suicide attempt and instead endorsed an aborted attempt or interrupted attempt. We classified these cases as having made a suicide attempt because the behaviors meet the consensus definition for an attempt. For example, one person that endorsed an aborted attempt (and denied a suicide attempt) reported taking an overdose of pills with an intention of dying, and subsequently lost consciousness but survived. Another reported drinking poison but then stopping halfway through the bottle, induced vomiting and calling poison control. These behaviors were coded as suicide attempts because they meet the consensus definition used in the field and because engaging in a potentially lethal behavior could result in death, even if a person subsequently changes his mind. For example, a person could drink poison, decide she does not want to die but lose consciousness before she is able to induce vomiting and die.

We trained coders to determine which suicide action the person had actually carried out based on their responses. Specifically, after establishing a codebook, coders practiced coding on training data generated by members of the research team (AJM, MDL) and pilot data not used in the final analysis. Each of four coders coded the dataset separately. Then each coder was assigned to one of two teams. The two teams independently came to a consensus code for each subject. The consensus codes from each team were then compared and evaluated for agreement. Coders determined whether there was enough information to classify the behavior into a suicidal category. For example, if when asked “What happened?”, a participant responded, “pills,” this would be coded as “not enough information to reclassify” and removed from the analysis. If the person responded, “I put the pills in my hand but at the last minute changed my mind and did not take any,” this would be considered to have provided enough information to classify that action (in this case as an “aborted attempt”). If at least one team of coders coded the participant’s action as “not enough information to reclassify,” the participant’s action was removed from analysis. Participants were also removed if the two coding teams disagreed on whether the action was a suicide attempt (total excluded; *n* = 84). If the coding teams agreed that there was sufficient information to code the entry and the action was not a suicide attempt but disagreed on the categorization of the action (e.g., one coding team said it was an aborted attempt and the other said the same entry was an interrupted attempt), it was coded as “Not an attempt; could not reclassify.” For entries with sufficient information, the coding process could result in the following final codes: (i) suicide attempt, (ii) interrupted attempt, (iii) aborted attempt (iv) no suicidal action (v) not an attempt; could not reclassify. There was high agreement among coders for determining whether an action met the definition for a suicide attempt (*Cohen’s k* = 0.87) and regarding whether there was enough information to determine that the action was not a suicide attempt (*Cohen’s k* = 0.85).

#### Preparatory Actions

Based each participant’s narrative description, coders determined the number of preparatory actions the person carried out. Due to difficulty measuring separate actions, the purpose of actions served as the main criterion. For example, a person that wrote two or ten suicide notes was still rated as having carried out one preparatory action of “communication.” We created a codebook with 27 different purposes; however, inclusion of some codes, automatically precluded others. For example, although travel to a location to make a suicide attempt and taking steps to prevent discovery could be separate actions, if a person traveled explicitly to prevent discovery, it was counted as a single preparatory action. We employed the same process described above where four coders: (i) were trained on responses generated by lead researchers and pilot data, (ii) coded items separately, (iii) joined teams of two, and determined a consensus number of preparatory actions for each participant. Each team’s consensus codes were compared and in instances where the number of coded preparatory actions differed, the two were averaged. Examples of codes included: actions to prepare for the suicide attempt (e.g., researching methods to attempt suicide), or actions to prepare for the fact that the person might be dead soon (e.g., writing a suicide note). Coders had a high degree of agreement (intraclass correlation: 0.96).

### Analyses

We calculated the percentages of people that endorsed each gate question. For ideation and attempt follow-up questions, participants were classified based on their most severe thought or action (e.g., active ideation overruled passive ideation). For each suicidal outcome, we calculated the percentage of people that endorsed a follow-up question or were coded as carrying out a particular action, among those that either endorsed or denied the gate question. The validity of the suicide ideation and planning gate questions were evaluated with follow-up responses, whereas the suicide attempt gate question was validated with coded actions. To demonstrate the classification properties of the gate and follow-up questions, we calculated sensitivity, specificity, positive predictive value and negative predictive value statistics. We include classification properties of gate and follow-up questions among the entire samples, as well as among suicidal ideators.

#### Statistical Simulation

We conducted a statistical simulation to understand whether, and to what extent, misclassification of suicide outcomes increases the probability of erroneous statistical results in studies of such outcomes. Statistical simulations model statistical inference by drawing samples from populations with specific parameters (e.g., two normal distributions with specified means and standard deviations). The current simulation drew samples from two different normally distributed populations (the “true effect” simulation) symbolizing two groups (e.g., non-attempters and suicide attempters). To represent misclassification, we placed data points from one sample in the other sample and vice versa based on the degree of misclassification that we actually observed in the current study. By sampling and then conducting between-groups t-tests 50,000 times and calculating the percentage of significant results (i.e., *p* < .05), we could examine whether, compared to normal conditions, misclassification reduced the probability of detecting the true effect. We also conducted an identical simulation with one exception–the samples were drawn from a single population, (the “true null effect” simulation) and we tested whether misclassification increased the probability of finding a significant effect when one did not truly exist.

**Fig 2:**
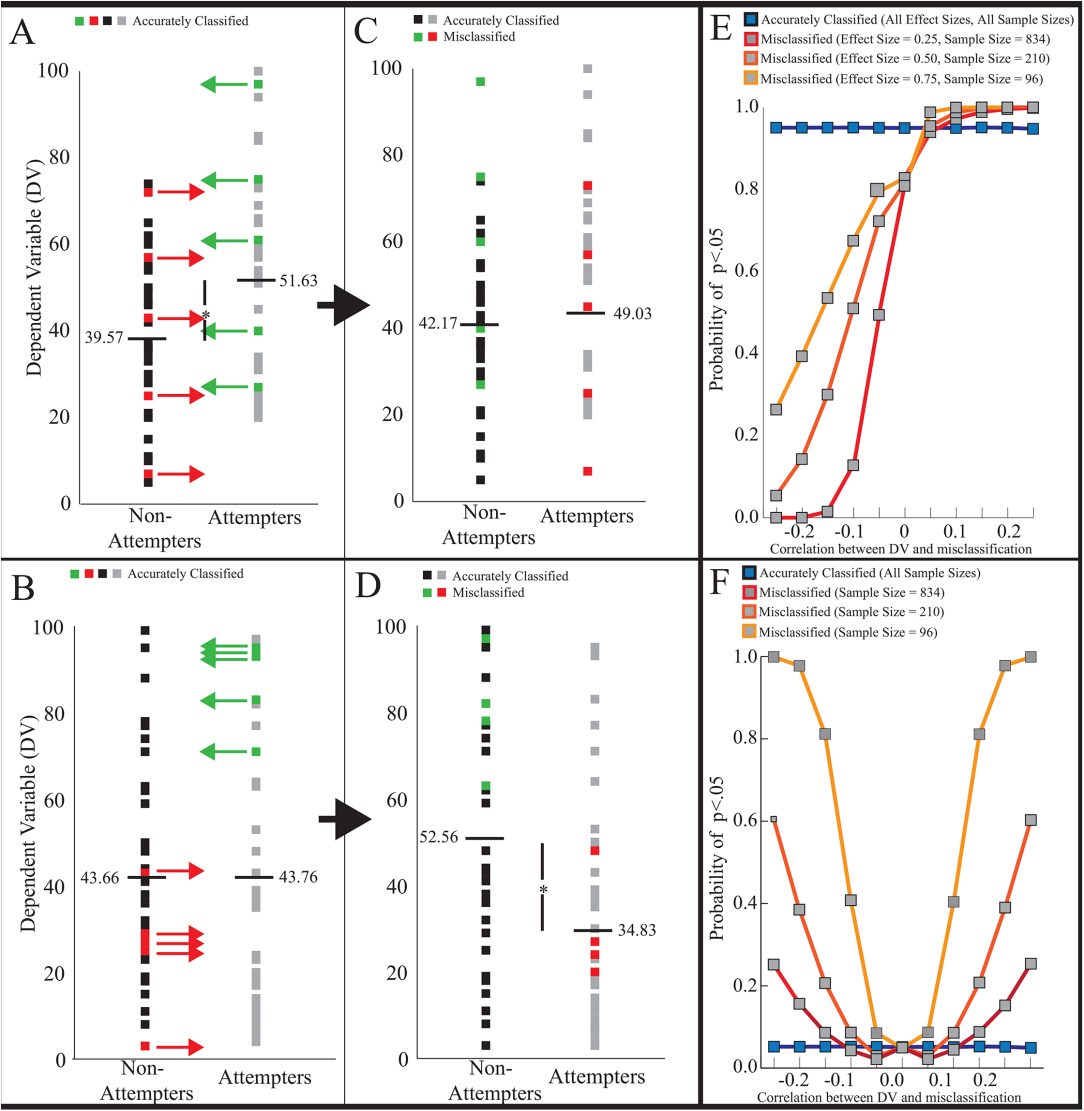
A visual example of DV-misclassification relationships and the results of the statistical simulations. (A) Model data of a “true effect" (B) Model data of a “true null effect.” The red and green arrows in (A) and (B) represent data points that will be misclassified due to participants inaccurately categorizing their suicidal behavior. (A) and (C) show an example of when the misclassification is random (i.e., dependent variable (DV)-misclassification correlation is zero). Because true attempters tend to be higher on the DV, true attempters randomly misclassified into the non-attempters group will tend to increase the group mean of non-attempters (e.g., from 39.57 in (A) to 42.17 in (C)). Likewise, in the example, true non-attempters tend to be lower on the DV and therefore, true non-attempters randomly misclassified into the attempters group will reduce the group mean of attempters (e.g., from 51.63 (A) to 44.03 in (C)). Thus, when there is a true difference between the groups at the population level, random misclassification will cause the two group means to move closer together reducing the power to detect the true effect. (B) and (D) shows an example of when there is a pattern to the misclassification (the DV-misclassification correlation is approximately *r* = 0.20). In this example, attempters higher on the DV tend to misclassify their behavior, causing an increase in the mean of non-attempters (e.g., from 43.66 in (B) to 52.56 in (D)). Non-attempters that are lower on the DV tend to misclassify their behavior, causing a decrease in the mean of attempters (e.g., from 43.76 in (B) to 34.83 in (D)). Thus, during a “true null effect” when the DV-misclassification correlation is present, misclassification causes the group means to shift further apart, increasing the probability of finding a false significant difference. (E) The outcome of the statistical simulation for data with a “true effect” across different effect sizes (varying sample sizes to maintain statistical power at 0.95). Without a DV-misclassification correlation, power to detect the true effect is reduced by approximately 15% across all effect sizes. Power decreases as the DV-misclassification correlation strengthens in the opposite direction of the true effect, increasing the chance of a false negative result (i.e., Type II error). This reduction in power is greater when the effect size is small and the sample size is large compared to when there are larger effect sizes and smaller sample sizes. (F) The “true null effect” across the same sample sizes as in the true effect simulation (alpha = 0.05). When misclassification is random for a “true null effect,” it does not affect the false positive error rate. As the DV-misclassification correlation strengthens in either direction, chances of a false positive increase (i.e., Type I error). Compared to larger sample sizes, chance of a false positive is greatest when there is a smaller sample size.

A parameter in the simulation, namely, the relationship between the dependent variable (DV) and misclassification itself, requires elaboration. To illustrate this idea, perhaps non-attempters with higher emotional reactivity are more likely to misclassify themselves as having attempted suicide because they felt so close to attempting. If this were the case, then there would be a relationship between the DV (emotional reactivity) and a tendency to misclassify one’s behavior (see Fig 2A–2D for visual example). Although this relationship is unclear in studies that assume accurate classification, it is unlikely to be exactly zero. Therefore, to examine the statistical effects of misclassification at different levels of a DV-misclassification relationship, we forced the simulated samples to correlate with misclassification (*r* values ranging from -0.25 to 0.25 by increments of .05).

For simplicity, when the simulation included two populations, mean differences were set to 0.5, 1.0, and 1.5, with a standard deviation of 2.0 to compare the simulation at large, medium and small effect sizes (Cohen’s *d*s of 0.25, 0.50, and 0.75, respectively) [12]. Statistical power was maintained at 0.95 across the three different effect sizes resulting in different total samples sizes (total sample size of 96, 210 and 834 for the large, medium and small effect size simulations, respectively). For consistency and to observe the effect of misclassification on a null effect with different sample sizes, we set the same standard deviation and sample sizes when drawing from a single population but there is no effect size when no group differences exist. Each of the 66 simulations (true and null effect simulations at the 3 differing effect sizes/sample sizes, each at 11 *r* values) was permuted 50,000 times. The details regarding computer code for the statistical simulation is located in S1 Materials.

## Results

### Suicide Ideation

In total, 78.9% of all participants endorsed the suicide ideation gate question (Table 2). Of those participants, only 74.6% endorsed the most extreme follow-up question “I should kill myself.” 16.5% indicated that their most extreme suicidal thought was the less certain response of “maybe I should kill myself,” with the other 8.8% reporting, at most, passive suicidal thoughts (e.g., “I wish I was dead”). Among those who denied suicidal ideation in the gate question, 3.3% endorsed the most extreme follow-up question, and another 10.5% indicated that they had the thought “maybe I should kill myself,” and approximately one-third (38.3%) reported passive suicidal thoughts ranging from “I wish I was dead” to “I wish I could disappear or not exist.” See Table 2 for all results.

**Table 2:** Responses to gate and follow-up questions for suicide ideation.

A. Gate Question: Suicide Ideation	YES		NO	
"Have you ever seriously thought about killing yourself?" (n = 1570)	%	Num	%	Num
	78.85	1238	21.15	332
**Follow-Up Questions (Most Severe)**	Among those who said YES to Gate		Among those who said NO to Gate	
"I should kill myself"	74.64	924	3.31	11
"Maybe I should kill myself"	16.56	205	10.54	35
"I wish I was dead"	5.57	69	16.87	56
"My life is not worth living"	0.97	12	8.43	28
"I wish I was never born"	0.24	3	3.01	10
"I wish I could disappear or not exist"	0.81	10	9.94	33
"What happens to people when they die?"	0.89	11	34.64	115
"What will it be like when I die?"	0.08	1	6.33	21
"No to all follow-up questions"	0.24	3	6.93	23

### Suicide Planning

In total, 33.1% of ideators without a history of suicidal actions endorsed the suicide plan gate question (Table 3). Among these participants, 93.1% thought of a method, 75.9% thought of a place, and nearly half (48.8%) had engaged in preparatory actions. A sizeable portion of those with ideation who *denied* ever making a suicide plan reported engaging in behavior that could be thought of as planning. For instance, 64.7% reported that they thought of a suicide method, 38.1% thought of a place, and 29.2% actually engaged in preparatory actions. A fairly large percentage of both those endorsing a plan (61.5%-64.2%) and those denying a plan (37.7%-40.9%) reported being sure of their method and place, and a sizeable portion of both groups (39.9%-51.7%) endorsed at least four of the items in Table 3.

**Table 3:** Responses to gate and follow-up questions for suicide plans.

B. Gate Question: Suicide Plan	YES		NO	
"Have you ever made a plan to kill yourself?" (n = 263)	%	Ratio	%	Ratio
	33.08	87/263	66.92	176/263
**Follow-Up Questions** ^**a**^	Among ideators who said YES to Gate		Among ideators who said NO to Gate	
Thought of a method	93.10	81/87	64.77	114/176
Thought of a place	75.86	66/87	38.07	67/176
Preparatory actions	48.75	39/80	29.19	47/161
Sure of method	64.20	52/81	37.72	43/114
Sure of place	61.54	40/65	40.91	27/66
Four or five planning components^b^	51.72	30/58	39.50	18/48

^a^ Participants who responded “No response/Not applicable” or left the item blank were excluded resulting in changing totals (i.e., denominators).

^b^ Only participants that endorsed or denied all 5 planning components were included. Using the full sample in the denominator (i.e., 176), 10.80% of people who denied a plan, endorsed four or five planning components

**Fig 3:**
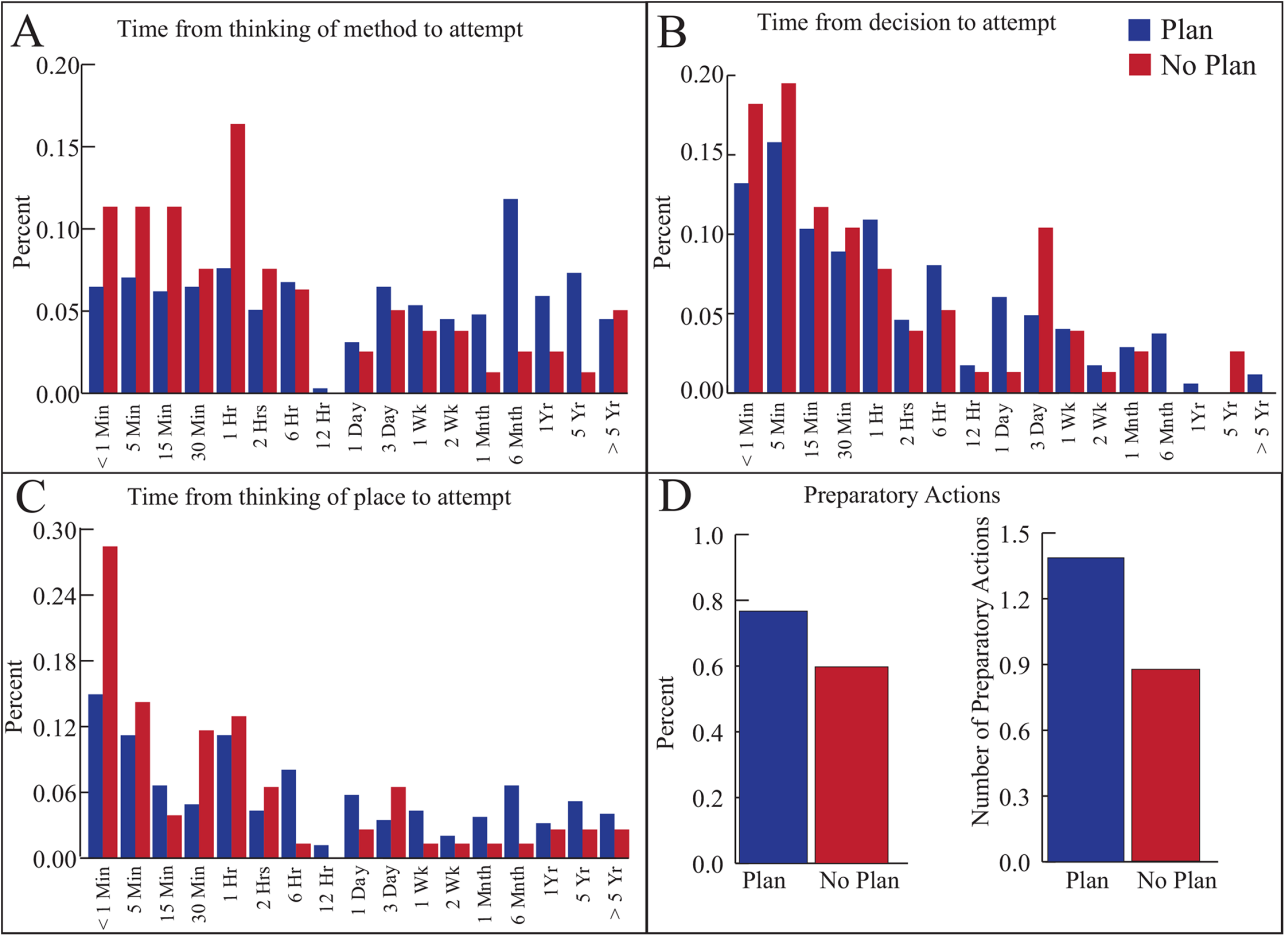
Characteristics of suicide planning among participants who attempted suicide and either endorsed a single-item plan or did not. For those that endorsed a single-item lifetime suicide plan (blue) and for those that denied a single-item lifetime suicide plan (red), the distributions of (A) the amount of time between thinking of the method and attempting suicide, (B) the decision to attempt and attempting suicide and (C) thinking of place to attempt suicide and attempting suicide and (D) the percentage of participants that made *any* preparatory actions and the mean number of preparatory actions prior to a suicide attempt. If participants interpreted a “suicide plan” similarly, one would expect minimal overlap between distributions of planning characteristics for single-item “planners” and “non-planners.” Instead, each of the distributions overlap substantially (71–92%) using the bins in the current plot, suggesting participants do not reliably interpret the single-item suicide planning question.

Examination of the data on suicide planning among attempters reveals several interesting results (Fig 3). For all participants the median amount of time prior to attempting that people thought of a method and thought of a place was 6 hours and 1 hour, respectively. The median amount of time between making a decision to kill oneself and making an attempt was 30 minutes. The mean number of preparatory actions was 1.3. Those who did not endorse having made a suicide plan reported moving quicker from thought to action (Fig 3A–3C) and making less preparatory actions (Fig 3D); however, when examining time estimates between those who endorsed a suicide plan and those who did not, there was substantial overlap (71–92% using the bin sizes in Fig 3A–3D) between planners and non-planners on each characteristic.

### Suicide Attempts

**Fig 4:**
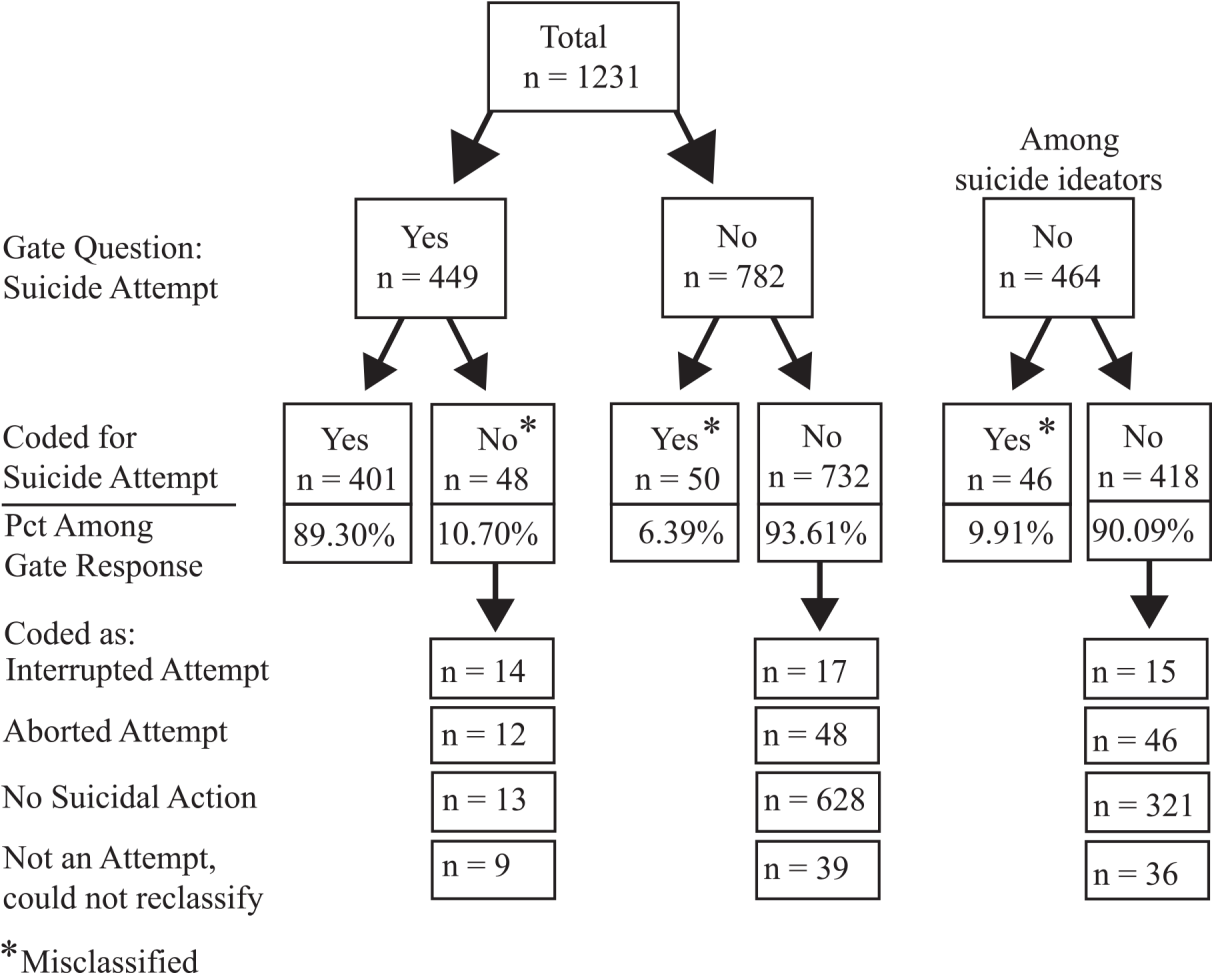
Responses to single-item suicide attempt question and the results of coded narrative responses. Single-item suicide attempt questions result in a 10.7% false positive rate and 6.39% false negate rate for the sample (9.91% false negative rate among ideators).

Overall, 36.6% of participants endorsed the suicide attempt gate question (Fig 4). Of those participants, 89.3% were determined by coders to have actually made a suicide attempt, whereas 10.7% were determined to have not made an attempt.

In contrast, 6.4% of all participants (and 9.9% of ideators) who denied the suicide attempt gate question were determined to have made a suicide attempt. An additional 8.3% of those who did not endorse the suicide attempt gate question were determined to have engaged in either an interrupted or aborted suicide attempt.

We also used the attempt coding to evaluate the follow-up attempt questions to determine whether embedding definitions in the questions and providing participants with several response options (i.e., increasing *clarity* and *coverage*) reduced misclassification. We found that the percentage of people that incorrectly endorsed attempting suicide dropped from 10.7% (using the gate question) to 4.8% (using the follow-up questions). Providing a broader range of response options also lowered the percentage of false negatives among the entire sample (4.2% versus 6.4% for the gate question) and among ideators (7.9% versus 9.9% for the gate question).

### Statistical simulation

The results of the statistical simulation are presented in Fig 2E and 2F. Results revealed that during the true effect simulation with a medium effect size, when data were randomly misclassified (i.e., DV-misclassification correlation was zero,), misclassification causes an approximately 15% drop in power to detect a true effect (i.e., Type II error). Under a true effect, if the DV-misclassification correlation is in the same direction as the effect, it slightly increases the probability of a significant result, leading to a trivial overestimation of a true effect. However, if the DV-misclassification correlation is in the opposite direction of the true effect, as the relationship grows stronger, the effect is increasingly underestimated, raising the probability of Type II error (Fig 2E). In this circumstance, a small effect size (and large sample size) increases the chance of Type II error compared to medium and large effect sizes (Fig 2E).

In the “true null effect”, when there is a small (*r* < ± 0.05) or no DV-misclassification relationship, the probability of rejecting the null hypothesis when no effect truly exists (i.e., a Type I error) remains intact. However, as the DV-misclassification relationship increases in either a positive or negative direction it rapidly escalates the probability of Type I error. For example, when the DV-misclassification relationship is *r* = ± 0.10 with a medium effect size, one will erroneously reject the null hypothesis 9% of the time (versus the typically acceptable 5%) but 20% of the time when *r* = ± 0.15 (Fig 2F). When the sample size is smaller, the increase in Type I error is more dramatic compared to when sample sizes are larger.

### Classification Statistics

The classification statistics for the suicide attempt gate question are contained in Table 4. Overall, the single-item gate question for suicide attempts demonstrated strong sensitivity and specificity, as well as positive and negative predictive value. However, the follow-up questions demonstrated improved numbers across every classification statistic within the entire sample and among suicidal ideators.

**Table 4:** Classification statistics for the suicide attempt gate and follow-up questions.

	Gate Question		Follow-Up Question		Gate QuestionAmong Ideators		Follow-up Question Among Ideators	
**Sensitivity**	0.89	(401/451)	0.93	(418/451)	0.90	(393/439)	0.94	(371/395)
**Specificity**	0.94	(732/780)	0.97	(759/780)	0.91	(418/461)	0.95	(278/293)
**PPV** ^**a**^	0.89	(401/449)	0.95	(418/439)	0.90	(393/436)	0.96	(371/386)
**NPV** ^**b**^	0.94	(732/782)	0.96	(759/792)	0.90	(418/464)	0.92	(278/302)

^a^ PPV = Positive Predictive Value

^b^ NPV = Negative Predictive Value

Note: Sensitivity is the proportion of actions coded as a true suicide attempt among people that endorsed a suicide attempt on the gate and follow-up questions. Specificity is the proportion of behaviors coded as not being a suicide attempt among people that denied a suicide attempt on the gate and follow-up questions. Positive predictive value is the proportion of people that endorsed a suicide attempt on the gate and follow-up questions among those that were coded as having attempted suicide. Negative predictive value is the proportion of people that denied a suicide attempt on the gate and follow-up questions among those that were coded as not having attempted suicide. Raw values for the proportions are given in parentheses.

## Discussion

There are four key findings in this study. First, this study revealed that the use of single-item measures of suicidal thoughts and behaviors is associated with a fair degree of misclassification. Critically, for suicide attempts, we rigorously coded participants’ narrative descriptions to provide careful assessment of misclassification. Second, single-item measures fail to capture a large range of potentially important distinctions in suicidal behaviors, such as passive ideation, various suicide planning steps and stopped suicidal actions. Third, statistical simulations revealed that the level of misclassification observed among single-item measures (11% false positive rate and 10% false negative rate among ideators) can substantially increase the probability of false conclusions from statistical tests. Fourth and finally, follow-up questions were associated with lower misclassification rates. These results suggest that single-item questions lack clarity and coverage and that one partial solution to the problems revealed in this study is to (i) embed the definition of the assessed behavior in the question and (ii) provide multiple response options. Several aspects of these results warrant further comment.

The misclassification produced by single-item assessment suggests that a considerable number of participants do not share researchers’ definitions for suicidal outcomes and single-item questions do not clearly inform these participants of this definition. Further supporting this conclusion, follow-up questions with more specific language had lower rates of misclassification, although the increased number of response options might have also contributed to the improved accuracy.

We also found that participants that endorsed single-item questions did not have homogenous thoughts or behaviors. For example, one might expect that people that “seriously thought about killing themselves,” means they had the thought, “I should kill myself.” However, we found that approximately 9% of the people who endorsed the former item never experienced active suicidal ideation. These results suggest that single-item questions do not cover the full range of suicidal thoughts and behaviors and people with heterogeneous behaviors are categorized as a homogenous group (e.g., “ideators”). The lack of response options may cause single-item measurement to obscure important associations or leave participants that engaged in omitted behaviors (e.g., aborted attempt) unsure of where to classify their behavior, increasing the likelihood of misclassification. It is important to note that several newer instruments, such as the Columbia-Suicide Severity Rating Scale (C-SSRS; [13]) assess a fuller range of suicidal behaviors, including aborted and interrupted attempts. However, the C-SSRS (and similar instruments) (i) requires a trained interviewer to administer and (ii) has been used in considerably fewer studies than single-item measures.

Distinguishing between aborted, interrupted and actual suicide attempts is important to advancing the understanding of suicide for two reasons. First, prior suicide attempts are the largest predictor of a future attempt [14,15]; however, the extent to which aborted and interrupted attempts predict future behaviors is unknown because they are rarely measured. This knowledge could help inform clinicians’ assessment of suicide risk. Second, there might be different risk factors for the different behaviors. For example, prior research suggests that increased impulsiveness is a risk factor for attempting suicide [16]. However, one would expect that inhibiting an urge to attempt suicide after taking steps to do so, as occurs in an aborted attempt, might be related to *reduced* impulsiveness. Understanding how unique risk factors play a role in different suicidal behaviors (e.g., aborted attempts versus attempts) could provide insight into the causes of suicide and improve prevention. Critically, when attempters are grouped together with non-attempters it is more difficult to identify the factors that influence people to actually engage in potentially lethal behaviors with the purpose of dying.

It could be argued that interrupted attempts (e.g., a police officer tackling someone about to jump off a bridge) should be combined with actual attempts because the person would have attempted suicide had they not been stopped. This reasoning is problematic for two reasons. First, given that people report stopping their action just prior to attempting, one cannot assume that approaching a suicidal action is the same as actually engaging in a potentially harmful behavior. Second, if future studies find that characteristics and risk factors of interrupted attempts match those of actual attempts, then it might be appropriate to combine these categories. However, researchers first need to distinguish among interrupted attempts and suicide attempts in order to make that determination.

Researchers often create groups based on single-item questions [6,17,18]. We found single-item assessment leads to inaccurate group categorization. For example, some participants coded as having attempted suicide would be placed in a “non-attempters group” based on their response to a single-item question. This would result in some actual attempters categorized as attempters but others categorized as non-attempters, eroding group distinctions. Maintaining accurately classified groups is crucial because, in clinical research, group membership is the basis for determining relationships between suicidal behaviors and important risk factors or psychological variables. When people in different groups have actually had identical thoughts or behaviors, categorical differences are no longer present undermining efforts to understand suicidal behaviors. Notably, the current study found misclassification among attempters and non-attempters in fairly equal numbers. If true in the general population, misclassification may not affect base rates but would adversely affect the ability to detect associations between important risk factors and suicide.

On one hand, the single-item question for suicide attempts showed fairly high accuracy and classification properties. This is consistent with a prior study examining single-item questions for suicide attempts [10]. Compared with this prior study, the current study makes several new additions evaluating single-item questions for suicide attempts, including (1) rigorously coding narrative descriptions to evaluate the questions of interest, (2) the addition of a statistical simulation to further evaluate the effect of misclassification on research studies and (3) testing the accuracy of a new approach to improve classification.

Despite the fairly strong accuracy for single-item questions, the results of the statistical simulation suggest that misclassification at the levels observed in this study can lead to both false positive and false negative statistical conclusions in research studies. The simulation also revealed that a true large effect size results in the lowest chance of a false conclusion; however, the null effect simulation revealed the smaller sample size required to detect a large effect is more susceptible to a false positive conclusion. Generally, the simulation suggests that larger effect sizes and larger samples will reduce false conclusions but preventing or reducing misclassification will further decrease the chance of statistical errors. The simulation results also suggest that prior studies that contain single-item questions may have, for example, shown support for relationships between a suicidal behavior and another variable that does not exist or, alternatively, failed to detect a relationship that does. Researchers cannot have confidence in a prior literature that might contain multitudinous statistical errors. For this reason, it is imperative that future work continues to evaluate single-item measurement and, if misclassification persists, researchers stop using this methodology and instead use items with multiple response options where the questions contain definitions for the behaviors being assessed.

We found that adding multiple response options and embedding the definitions of specific behaviors within the question (i.e. the follow-up questions) improved accuracy and showed stronger classification properties compared with single-item questions. This provides researchers with a fairly simple modification to improve (though not perfect) the classification of the measurement of suicide attempts. The disadvantage of multiple questions is that it may take participants a few minutes longer to complete. However, we believe that more accurate measurement is worth the additional time. In addition to improved accuracy, the multiple response options provides researchers an opportunity to study other suicidal behaviors, such as aborted and interrupted attempts, that barely have been examined. Better understanding these behaviors could improve understanding of suicide as well as prediction and prevention efforts.

Several of our findings regarding suicide planning warrant additional comment. First, the terms “suicide plan,” “planned attempt” and “impulsive” or “unplanned attempt” remain poorly defined [19]. If researchers have not agreed on an operational definition of a suicide plan or a planned attempt, participants are unlikely to hold reliable or valid conceptualizations of these terms. For example, in a prior study more than a quarter of attempters gave paradoxical descriptions of their attempts as either (i) “impulsive” but “planned” or (ii) “not impulsive” but with no “planning” [20]. Similarly inconsistent, in the current study, substantial numbers of ideators who denied planning during the gate question had engaged in several planning steps (e.g., thinking of a method and/or place to attempt suicide). Second, this study used novel methods to collect the timing of several planning items prior to a suicide attempt and examined the entire distribution for these items. In general, the majority of planning occurs within a day. Some individuals move through the planning stages quickly (< 5 min) whereas others do so over weeks and months. Future research should continue to examine suicide planning to better understand the critical time period when people transition from thinking about suicide to actually attempting.

There are several limitations to this study. First, the sample is not representative and over-represents females and clinical/suicidal people compared with the general population. Second, most of the assessment items used relied on retrospective recall of suicidal thoughts and behaviors and memories of events may be subject to bias or inaccuracies. Third, we excluded participants from analyses of suicidal actions because coders were unable to identify their exact suicidal behavior. The results of the study may have been altered if we had interpretable data from these participants. Fourth, the data were collected over the internet and therefore could have affected by lack of earnest responses. However, we rigorously inspected the data and removed participants with nonsensical responses and several studies have demonstrated the validity of internet responses [21]. Despite these limitations, the results suggest that single-item questions lead to inaccurate responses that impede progress describing, understanding, and ultimately preventing suicidal behaviors.

## Conclusion

We found that single-item questions regarding suicide ideation, plans and attempt can cause misclassification and, under particular circumstances, can increase the probability of both Type I and Type II statistical errors. Therefore, the single-item approach might be impeding scientific progress understanding and preventing suicide. In addition, people that endorse single-item suicidal behaviors have actually engaged in a variety of thoughts and behaviors that are neglected by the single-item approach. Providing multiple response options for people to place suicidal behaviors and embedding definitions for these behaviors in the questions reduced both false positives and false negatives; however, these question still contained misclassified responses. Future research should continue to refine and test methods that maximize participants’ accurately categorizing their behaviors in order to improve the understanding and prevention of suicide.

## Supporting Information

S1 Materials Information regarding study materials including a link to download dataset, study materials and analysis code. (DOCX)

S1 Online Survey Tables A-E contain the precise wording of several questions in the survey used in the current study. (DOCX)
