# Supplementary material for: Single-Item Measurement of Suicidal Behaviors: Validity and Consequences of Misclassification
Source: PLoS One. 2015 Oct 23;10(10):e0141606. doi: 10.1371/journal.pone.0141606 (PMC4619664; doi:10.1371/journal.pone.0141606)
Supplement: S1 Materials — (DOCX) [file pone.0141606.s001.docx]

**S1 - Materials**

The following are available for download: (1) A Qualtrics file and Word document containing the full version of the online survey, (2) instructions, dataset, dataset guidebook and computer code that will reproduce results for all analyses, (3) computer code producing the statistical simulation. These are available at http://dx.doi.org/10.17605/OSF.IO/Q36BG.

The simulations were conducted using Python (version 2.7.9) packages numpy (version 1.9.0; for mathematical analysis), scipy (0.14.0; for mathematical and statistical functions) and pandas (version 0.14.1; dataframes to manage and store data).
