# Supplementary material for: Single-Item Measurement of Suicidal Behaviors: Validity and Consequences of Misclassification
Source: PLoS One. 2015 Oct 23;10(10):e0141606. doi: 10.1371/journal.pone.0141606 (PMC4619664; doi:10.1371/journal.pone.0141606)
Supplement: S1 Online Survey — (DOCX) [file pone.0141606.s002.docx]

**S1 - Online Survey**

For precise wording of online survey questions, see S2 Tables A-E. The questions in S2 Table D and Table E are from the follow-up section for a participant that endorsed multiple suicide attempts. Follow-up questions for participants that endorsed a single suicide attempt or a different suicidal action are similar but worded slightly differently. S1 – Materials contains a link to the Qualtrics file and Word document containing the entire survey. Please seek authors’ approval prior to collecting data with these instruments.

| These questions apply to your entire life, including a long time ago or when you were a child.^a^ |
| --- |
| Have you ever seriously thought about killing yourself? |
| Have you ever made a plan to kill yourself? |
| Have you ever attempted to kill yourself? |

**S2 Table A. Gate instructions and gate questions**

^a^Possible responses were (1) Yes (2) No

| The next series of items will be similar to the previous questions. Please answer as accurately as possible, regardless of your responses to previous questions.^a^  These questions apply to your entire life, even when you were a child or a long time ago.  Please read each question carefully.  Have you ever thought of any of the following for longer than a few minutes (more than passing thoughts): |
| --- |
| What happens to people when they die? |
| What will it be like when I die? |
| I wish I could disappear or not exist |
| I wish I was never born |
| My life is not worth living |
| I wish I was dead (for example go to sleep and not wake up again) |
| Maybe I should kill myself |
| I should kill myself |

**S2 Table B. Follow-up instructions and follow-up questions for ideation**

^a^Possible responses were (1) Yes (2) No (3) Prefer not to respond

**S2 Table C. Follow-up instructions and follow-up questions for actions**

| Please answer as accurately as possible, regardless of your responses to previous questions.^a^  These questions apply to your entire life, including a long time ago or when you were a child.  Select one button for each time something happened. If you think one incident falls into multiple categories pick the one that best describes what happened.  Please read each question carefully.  Have you ever: |
| --- |
| Done something to hurt yourself on purpose without intending to die? (e.g., cutting yourself, hitting yourself, or burning yourself) |
| Done something to make it look like you were trying to kill yourself, when you did NOT intend to die? (e.g. let people know that you were in distress, get revenge or a reaction) |
| Started to take steps to kill yourself and, at the last minute, you stopped because you decided not to kill yourself? (e.g., (1) you went to a bridge but did not jump off, (2) took out pills but did not take them, (3) took out a gun but did not pull the trigger) |
| Started to take steps to kill yourself and, at the last minute, someone or something else stopped you? (e.g., (1) you went to a bridge but someone stopped you before you jumped off, (2) took out pills but someone stopped you before you took them, (3) took out a gun but someone stopped you before you pulled the trigger) |
| Tried to kill yourself; meaning you engaged in a potentially deadly behavior to end your life with some intention of dying? (e.g., (1) you went to a bridge and jumped off, (2) took out pills and you took them, (3) took out a gun and pulled the trigger) |

^a^Possible responses were (1) Yes (2) No (3) Prefer not to respond

**S2 Table D. Questions for planning prior to a suicide action**

| When asked how you tried to kill yourself, you said: {Previously Entered Method}    How long was it from the first time you thought of this method to when you actually tried to kill yourself? (It could be any amount of time; such as 5 years, 3 months, 4 weeks, 2 days, 3 hours, 4 minutes or 30 seconds)^a^ |
| --- |
| When asked where you tried to kill yourself, you said: {Previously Entered Place}  How long was it from the first time you thought of this as a place where you could kill yourself to when you actually tried to kill yourself? (It could be any amount of time; such as 5 years, 3 months, 4 weeks, 2 days, 3 hours, 4 minutes or 30 seconds) ^a^ |
| "Mulling over" is when you are very strongly considering killing yourself or, perhaps, going back and forth in your mind about whether you should actually do it or not. When you are mulling, you are close to trying to kill yourself but have not yet decided that you will try.  How long did you mull over the decision to try to kill yourself? (It could be any amount of time; such as 2 days, 3 hours, 4 minutes or 30 seconds)^a^    If you never mulled over your decision, enter a 0 |
| How long was it from the time you decided you were going to try to kill yourself to when you actually tried to kill yourself? (It could be any amount of time; such as 2 days, 3 hours, 4 minutes or 30 seconds)^a^ |
| Think back to the most recent time you tried to kill yourself.  Beforehand, did you do anything to prepare for the suicide attempt?^b^  Did you: try to get away from other people, check your insurance, clean up your house or apartment, give things away, make a will, say goodbye to people, or write a suicide note, or do anything like that to prepare for the attempt or because you thought you might be dead soon?  Please list all actions below: |
| You said, the most recent time you tried to kill yourself, you used:{Previously Entered Method}  Perhaps, at first you had only a rough idea that you would use this method but then, at some point, you were SURE you would use this method if you tried to kill yourself.  How long was it from the point you were SURE you would try to kill yourself using this method to when you attempted to kill yourself? (It could be any amount of time; such as 5 years, 3 months, 4 weeks, 2 days, 3 hours, 4 minutes or 30 seconds)^a^ |
| We apologize if you included this in your answer regarding preparation  Did you have to do something to obtain this method (e.g., go out of your way to get pills or a weapon)?^c^ |
| When you were obtaining this method, were you intending to use it to kill yourself or was it obtained for some other reason?^d^ |
| The most recent time you tried to kill yourself, you were: {Previously Entered Place}  Perhaps, at first you had only a rough idea that you would be in this place but then, at some point, you were SURE you would try to kill yourself in this place.    How long was it from the point you were SURE you would try to kill yourself in this place to when you attempted to kill yourself? (It could be any amount of time; such as 5 years, 3 months, 4 weeks, 2 days, 3 hours, 4 minutes or 30 seconds)^a^ |
| We apologize if you included this in your answer regarding preparation  Did you have to travel to this place?^c^ |
| Did you travel with the goal of going somewhere to kill yourself?^e^ |

^a^Responses consisted of a text box to enter a number and a drop-down list containing the time units listed in the question.

^b^Participants could respond in a free response text box

^c^Possible responses were (1) Yes (2) No (3) Other (please explain)

^d^Possible responses were (1) Obtained to kill myself (2) Some other reason (3) Both. It was obtained for another reason, but I was also thinking I could use it to kill myself (4) Other (please explain)

^e^Possible responses were (1) Yes, I traveled to go to a place to kill myself (2) No, I was traveling for some other reason (3) Both. I was traveling for another reason but thought I might also try to kill myself (4) Other (please explain)

**S2 Table E. Follow-up questions to elicit narrative responses regarding the most recent suicidal incident^a^**

| Think back to the most recent time you tried to kill yourself.  What exactly happened? Please give as many specific details as possible about the period leading up to your decision to do this and what you did. For example, if you took pills, what kind of pills did you take and how many? |
| --- |
| Think back to the most recent time you tried to kill yourself.  Did you sustain any injuries or have any physical problems as a result? (e.g., internal injuries, such as stomach or other organ problems, a bruise, a headache, or a loss of consciousness)  Please describe below: |
| Think back to the most recent time you tried to kill yourself.  Why did you try to kill yourself? |

^a^All responses were free response text boxes
